# Consciousness, free will, and moral responsibility: Taking the folk seriously

**DOI:** 10.1080/09515089.2014.962018

**Published:** 2014-10-03

**Authors:** Joshua Shepherd

**Keywords:** Consciousness, Experimental Philosophy, Free Will, Moral Responsibility

## Abstract

In this paper, I offer evidence that folk views of free will and moral responsibility accord a central place to consciousness. In sections 2 and 3, I contrast action production via conscious states and processes with action in concordance with an agent's long-standing and endorsed motivations, values, and character traits. Results indicate that conscious action production is considered much more important for free will than is concordance with motivations, values, and character traits. In section 4, I contrast the absence of consciousness with the presence of consciousness in behaviorally identical agents. Most participants attribute free will to conscious agents, but not to nonconscious agents. Focusing in particular on two leading views of free will and moral responsibility, namely, Deep Self and Reasons-Responsive Views, I argue that these results present philosophers of mind and action with the following explanatory burden: develop a substantive theory of the connection between consciousness on the one hand and free will and moral responsibility on the other that takes folk views on this connection seriously.

## 1. Introduction

Philosophers who work on free will, moral responsibility, and related issues aim to take folk views on these issues seriously. There are at least two good reasons for this. First, arguments in favor of various theories of free will or moral responsibility often appeal to widely shared intuitions, and many philosophers take the fact that these intuitions are widely shared to lend them epistemic weight (see Sommers, 2010 for discussion). Second, our concepts of free will and moral responsibility are deeply embedded in our self-understanding. Theories of free will and moral responsibility that do not take folk views on them seriously would thus seem to be guilty of changing the subject. Nahmias and Murray put this point well:

‘Free will’ plays a central role in the conceptual scheme that we use to navigate the normative world via its connections to ‘moral responsibility’, ‘blame’, ‘autonomy’ and related concepts. Theorizing about ‘free will’ in isolation from the ordinary understanding of it thus risks being an academic exercise about some other, technical conception with understanding of it divorced from people's actual practices of assessing praise, blame, reward, and punishment, and from their understanding of themselves and their place in the world. (2014, p. 435)

This is not to say that folk views on free will and moral responsibility should determine the truth of theories about free will and moral responsibility. It is possible for competing considerations (e.g., those provided by arguments or by relevant empirical evidence) to undermine the epistemic weight of intuitions. Indeed, taking the folk seriously is compatible with a robust revisionism about free will—the view that “an adequate philosophical account of free will requires us to jettison some aspects of our commonsense thinking about it” (Vargas, 2009, p. 45). It is possible that the best theory of free will or moral responsibility will sacrifice at least some widely shared intuitions. At minimum, to take the folk seriously is to be aware of the relation between one's theory of free will and moral responsibility and relevant commonsense views, such that one is able to justify departures from common sense.^1^

In this paper, I report the results of three experiments that explore the connection between consciousness on the one hand and free will and moral responsibility on the other. I argue that given these results, two leading theories of free will and moral responsibility—or more precisely, of what is sometimes called moral responsibility-level free will^2^—fail, at present, to take the folk seriously. The experiments were motivated by three experiments I conducted and reported in Shepherd (2012). In those studies I presented participants with vignettes that contrasted the production of behavior by conscious processes with the production of behavior by nonconscious processes. When consciousness was involved in an agent's action production, participants attributed free will and moral responsibility to the agent. When consciousness was not so involved, most participants judged that the agent did not act freely or responsibly. These results are rather dramatic—varying the causal impact of consciousness is apparently enough to influence the attribution of free will and moral responsibility. Interestingly, this was true even in a case that varied not only the role of consciousness in action production, but also whether causation was deterministic or indeterministic (Shepherd, 2012, experiment 3). Although the presence of determinism did significantly impact ascriptions of free will, the effect size for consciousness was larger than that of determinism (2012, p. 924).

In light of these results, it is striking that the two leading theories of free will and moral responsibility pay little attention to the role of consciousness. Consider, first, so-called Deep Self Views, according to which an agent's free and responsible actions should bear some kind of relation to the features of the psychological structure constitutive of the agent's real self (Arpaly & Schroeder, 1999; Wolf, 1990). As Faraci and Shoemaker describe the structure of Deep Self Views,

the basic idea has been to identify a subset of an agent's motivating psychological elements as privileged for self-determination and responsibility, such that as long as one's actions are ultimately governed by this subset, they count as one's own and thus render one eligible for responsibility-responses to them. (2010, p. 320)

Deep Self theorists disagree about the nature and structure of the relevant psychological elements, but none of the precise formulations of the Deep Self View emphasize consciousness. Indeed, some Deep Self Views have been taken to directly oppose or undermine the intuitive role of consciousness for free and responsible action. Levy, discussing a collection of recent Deep Self theorists, notes the following:^3^

Most of these philosophers advance accounts of moral responsibility according to which agents are responsible for actions that are caused by and thereby *express* their propositional attitudes; it is not, they claim, necessary for an agent to be conscious of the attitudes his or her actions express for these actions to be appropriately expressive. (2011, p. 243)

Insofar as Deep Self theorists fail to include any place for consciousness in their theory, Shepherd's results represent a problem. In sections 2 and 3 of this paper, I sharpen the worry by presenting the results of two new experiments. In these experiments, I consider the possibility that an agent's conscious states and processes are relevant to folk views of free will primarily because they are connected to an agent's Deep Self. I directly contrast behavior produced by elements of an agent's Deep Self—that is, by elements of an agent's interior life (e.g., motivations, values, and convictions) that the agent clearly endorses—with behavior produced by an agent's conscious mental processes (or Conscious Self). Results indicate that though elements of an agent's Deep Self have a minor impact on judgments of free will, consciousness produces much larger effects. Given this, I argue that issues of psychological structure, considered in abstraction from consciousness, are insufficiently sensitive to folk views on free will and moral responsibility.

Consider, second, the Reasons-Responsive View. According to Fischer and Ravizza's well-known version of this view, an agent is morally responsible for an action only if that action is produced via a mechanism that both recognizes and reacts—in a sufficiently flexible way, and typically via action—to reasons for action (1998). There is much to like about this kind of view. But on the face of it, a Reasons-Responsive View highlights considerations orthogonal to consciousness. It is true that a connection between consciousness and reasons-responsiveness is sometimes *assumed* (Schlosser, 2013 is explicit about this). However, many leading Reasons-Responsive theorists rarely mention consciousness. Indeed, Yaffe claims that “there is no reason to suppose that consciousness is required for reasons-responsiveness” (2012, p. 182).

At the very least, it looks like there is work to do if a Reasons-Responsive View is to accommodate consciousness, or dismiss its importance as merely apparent. One natural way for a Reasons-Responsive theorist to accommodate consciousness would be to claim that consciousness is somehow functionally crucial for appropriate reasons-responding. In section 4, however, I present results that indicate that on the folk view, the connection between consciousness and free will and moral responsibility goes above and beyond functional considerations. If Reasons-Responsive theorists are to accommodate consciousness in a way that also accommodates the folk view, they may need to look beyond functional considerations.

The upshot of these experiments is that philosophers who care about free will and moral responsibility have work to do if they want to take folk views on free will and moral responsibility seriously. In section 5, I consider the theoretical landscape in this connection.

## 2. Experiment 1

### 2.1. Procedure

In this experiment, participants read one of four vignettes. This experiment utilized a 2 (Conscious Self: nonconscious versus conscious) ×  2 (Deep Self: concordant versus discordant) design that contrasted actions produced via conscious (or nonconscious) states and processes with actions produced in concordance (or discordance) with an agent's long-standing and endorsed motivations, values, and character traits (i.e., with an agent's Deep Self). Given the intuitive importance of both consciousness as well as an action's concordance with elements of the Deep Self, I predicted that both conditions would have a significant effect. That is, I predicted that free will and moral responsibility ratings would be highest in the conscious, Deep Self-concordant condition, lowest in the nonconscious, Deep Self-discordant condition, and in between in the other two conditions.

Participants in the concordant condition first read the following paragraph:

**Concordant**

Jim is a violent and aggressive man. He often instigates fights: he finds the prospect of fighting another man exciting. Jim clearly values this feature of his character. For example, he has the word ‘KILL’ tattooed on his fist, and he constantly brags to his friends about fights he instigates.

Participants in the discordant condition read the following paragraph instead:

**Discordant**

Jim is a peace-loving and docile man. He never instigates fights: he finds the prospect of fighting another man childish. Jim clearly values this feature of his character. For example, he donates a large percentage of his income to pacifist organizations, and he enjoys teaching conflict-resolutions seminars on weekends.

Then all participants read a paragraph that described Jim's unusual condition:

Jim has an unusual condition. Thanks to a neurological defect, Jim has no conscious awareness of the right side of his visual field. However, Jim clearly processes information from the right side of his visual field. We know this because when neurologists present an X or a Y to Jim's right side and ask him “Do you see an X or a Y?” Jim always gives the correct answer: even though Jim swears he is just guessing! Further, when neurologists present dots at various places on Jim's right side and ask him to touch the dots, Jim is very accurate. He is accurate even though, from Jim's perspective, it feels like he is just guessing at a random location. This is because Jim has no conscious awareness of the dots, or of anything on the right side of his visual field.

Participants then read a paragraph that sorted them into either a conscious or a nonconscious condition:

**Conscious**

Recently, Jim was at the bar playing darts for money, when he lost $100 to Larry, a man he hates [in the discordant condition, Larry was alternately described as “his friend”]. This made Jim very upset. Larry said, “I'm going to the bathroom. You better have my money when I get back.” As Larry returned, he crept up on what he wrongly thought was Jim's conscious blind side: his left side. Then he waved at Jim and silently sneered. Of course, since Larry was on Jim's left side, Jim was consciously aware that he was there. Suddenly, before Larry knew what had happened, Jim reached out and punched him—punched him hard—right in the nose.

**Nonconscious**

Recently, Jim was at the bar playing darts for money, when he lost $100 to Larry, a man he hates [in the discordant condition, Larry was alternately described as “his friend”]. This made Jim very upset. Larry said, “I'm going to the bathroom. You better have my money when I get back.” As Larry returned, he crept up on what he knew was Jim's conscious blind side: his right side. Then he waved at Jim and silently sneered. Of course, since Larry was on Jim's right side, Jim had no conscious awareness that he was there. Suddenly, before Larry knew what had happened, Jim reached out and punched him—punched him hard—right in the nose.

After reading one of these vignettes, participants rated their agreement to the following series of statements on a scale of 1–6, where 1 indicated “strongly disagree” and 6 indicated “strongly agree”:

**Free Will**

When Jim punched Larry, he did so of his own free will.

**Intentional**

Jim punched Larry intentionally.

**Moral Responsibility**

Jim is morally responsible for punching Larry.

**Decided**

Jim decided to punch Larry.

**Could Have Done Otherwise**

Jim could have decided not to punch Larry.

A potential worry with the vignette as formulated is that some participants might judge that though Jim could not consciously see Larry, he could nonetheless be consciously aware of Larry, or of what he was preparing to do (i.e., punch Larry) somehow. To test for this possibility, I also included the following statement for participants reading a vignette in which Jim does not consciously see Larry, and I asked participants whether the statement was true or false.

**Aware**

Jim was aware that he was punching Larry.^4^

### 2.2. Participants

Participants were recruited on Amazon's Mechanical Turk. 166 adults (82 male, 84 female) saw one of four vignettes.

### 2.3. Results

A 2 ×  2 analysis of variance (ANOVA) test was performed on participant responses to *Free Will*. I found a significant main effect for Conscious Self, F(1, 166) = 40.38, p <  .001, partial eta squared = .199, no effect for Deep Self (p = .15), and no significant interaction (p = .09). No matter the condition, most participants judged that Jim acted freely to some degree, and the effect was more pronounced when Jim was conscious (the mean responses by vignette were as follows: *Conscious*/*Concordant* M = 5.64, SD = .59; *Conscious*/*Discordant* M = 5.06, SD = .98; *Nonconscious*/*Concordant* M = 4.17, SD = 1.36; and *Nonconscious*/*Discordant* M = 4.21, SD = 1.32; see Figure 2). The mean responses (and patterns of statistical significance) for all other statements closely tracked responses to *Free Will*, and are thus not reported here.

Recall the above worry that some participants reading a nonconscious vignette might nonetheless find room for consciousness in the production of Jim's punch. Of the 99 participants who read a nonconscious vignette, 40 answered “true” (and 59 “false”) to *Aware*. Clearly many participants did not read the vignette as intended; they judged that consciousness was still in some way involved in the action. Moreover, this difference appeared significant. Participants who reported “false”—who thus read the vignette as intended—had a mean score of 3.85 (N = 59, SD = 1.30) in response to *Free Will*. Participants who reported “true” had a mean score of 4.68 (N = 40, SD = 1.23) to *Free Will*. A post-hoc independent samples t-test confirmed that this difference is significant (t(97) = 3.18, p = .002, two-tailed).

I was curious how participants understood *Aware*. Did their judgment that Jim was aware of punching Larry come with a judgment that Jim also decided to punch Larry? It seems so. Those who judged that Jim was aware of punching Larry also judged that he decided to do so (M = 4.45, SD = 1.20), while those who judged that Larry was not aware of punching Larry also judged that he did not decide to do so (M = 3.00, SD = 1.43). This difference is significant (t(97) = 5.29, p <  .001, two-tailed). Interestingly, a large portion—21/59, or 36%—of those who judged that Jim was not aware of punching Larry still judged that Jim decided to punch Larry (i.e., responded 4 or higher). The mean response to *Decided* for these participants was 4.52 (SD = .98).

### 2.4. Discussion

This experiment contrasted action production by way of the Conscious Self (that is, by way of conscious states and processes) with action concordant with the Deep Self (that is, concordant with long-standing and endorsed motivations, values, and character traits). As I noted in section 1, some Deep Self Views have been taken to directly oppose or undermine the intuitive role of consciousness for free and responsible action. Insofar as Deep Self theorists seek to downplay the importance of consciousness, these results give Deep Self theorists a reason to worry. The above vignettes contrasted cases in which Jim's action expressed his endorsed and stable motivations, values, and character traits with cases in which Jim's action was wildly out of character. But—contrary to my prediction—this made no significant difference to participant attributions of free will or moral responsibility. What did make a difference was the presence or absence of consciousness. Participants gave much higher free will ratings when Jim was conscious of Larry before he punched him, whether or not Jim's action was concordant with his Deep Self (M = 5.64 and M = 5.06). This indicates that the impact of consciousness on participant ascriptions of free will is independent of features often taken to motivate Deep Self Views.

These results do not present a *counterexample* to Deep Self Views. Participants attributed free will and moral responsibility to Jim even in the nonconscious conditions (M = 4.17 and M = 4.21), as many Deep Self Views would predict. Further, it is worth mentioning that though it is true that many Deep Self Views downplay the role of certain elements of conscious mental life, such as conscious choice or conscious control, this is not to rule out any role for consciousness in free, responsible behavior. Smith (2005), for example, downplays the importance of conscious choice in order to emphasize the importance of (passive, not necessarily conscious) evaluative judgment to our moral practices. But Smith's view seems to implicitly rely on elements of conscious mental life nonetheless. For we discern the nature of an agent's evaluative judgments, on Smith's view, in part by observing a number of these conscious elements: our spontaneous desires and emotions; the thoughts that “occur” in the conscious mind; our patterns of conscious attention; and so on. Similarly, Sher explicitly opposes what he calls the spotlight view, according to which “an agent's responsibility extends only as far as his awareness of what he is doing” (2009, p. 4). And yet Sher retains an important role for consciousness in his theory. Though he argues “we should think of each responsible agent not merely as a conscious center of will,” in the same paragraph he asserts that without also being a center of conscious thoughts and conscious deliberative activities, agents “would not qualify as responsible at all” (2009, p. 121).

Even so, these results suggest that the kinds of considerations taken to motivate Deep Self Views are relatively unimportant to laypeople when *compared* to the role of consciousness. In downplaying the importance of consciousness, such views contain a significant blind spot.

Some participants did not read the vignette as intended. Though told that Jim was not consciously aware that Larry was standing next to him, 40% of participants in the nonconscious condition judged that Jim was aware that he was punching Larry. These participants appear to have judged that even when conscious vision did not play a role, other conscious processes did. Further, many of these participants judged not only that Jim was aware of punching Larry, but that Jim decided to punch Larry (given the strong difference in responses to *Decided*, it is a plausible assumption that these participants did not have a nonconscious decision in mind). And even participants who judged that Jim was not aware of punching Larry appear unsettled about the judgment; the mean rating of 3.00 in response to *Decided* represents only mild disagreement. Further, 36% of participants who judged that Jim was not aware of punching Larry still judged that Jim decided to punch Larry. These participants apparently found it quite difficult to fully excise the thought that consciousness was somehow involved in the production of the punch. Experiment 2 was designed to avoid this difficulty.

## 3. Experiment 2

### 3.1. Procedure

A limitation of the previous experiment was that some participants found a role for awareness in the action even in nonconscious conditions. I designed this experiment to emphasize an even stronger distinction between action production via conscious processes and action production via nonconscious processes. Similar to experiment 1, this experiment utilized a 2 (Conscious Self: nonconscious versus conscious) ×  2 (Deep Self: concordant versus discordant) design. Given the results of experiment 1, I predicted that the Conscious Self condition would have a significant impact, and that the Deep Self condition would have at best a minimal impact.

Participants in the concordant condition first read the following paragraph:

**Concordant**

Jim is a violent and aggressive man. He often instigates fights: he finds the prospect of fighting another man exciting. Jim clearly values this feature of his character. For example, he has the word ‘KILL’ tattooed on his fist, and he constantly brags to his friends about fights he instigates.

Participants in the discordant condition read the following paragraph instead:

**Discordant**

Jim is a peace-loving and docile man. He never instigates fights: he finds the prospect of fighting another man childish. Jim clearly values this feature of his character. For example, he donates a large percentage of his income to pacifist organizations, and he enjoys teaching conflict-resolutions seminars on weekends.

Then all participants read a paragraph that described Jim's unusual condition:

Jim has an unusual condition. Thanks to a neurological defect, Jim has no conscious control over his left hand. And sometimes Jim's hand will do things Jim does not want it to. For example, when Jim is buttoning his shirt with his right hand, sometimes his left hand will start unbuttoning the shirt. Or when Jim is trying to raise a glass to his mouth with his right hand, Jim's left hand will knock the glass to the ground. Jim's condition is so odd because, though it seems like Jim's hand ‘knows what it is doing’, Jim cannot consciously control it.

Participants then read a paragraph that sorted them into either a conscious or a nonconscious condition:

**Conscious**

Recently, Jim was at the bar playing darts for money, when he lost $100 to Larry, a man he hates [in the discordant condition, Larry was alternately described as “his friend”]. This made Jim very upset. Larry sneered at Jim and said, “You better have my money.” Then Larry leaned towards Jim's face, chuckling. Suddenly, before Larry knew what had happened, Jim reached out with his right hand—the hand he can consciously control—and punched Larry—punched him hard—right in the nose.

**Nonconscious**

Recently, Jim was at the bar playing darts for money, when he lost $100 to Larry, a man he hates [in the discordant condition, Larry was alternately described as “his friend”]. This made Jim very upset. Larry sneered at Jim and said, “You better have my money.” Then Larry leaned towards Jim's face, chuckling. Suddenly, before Larry knew what had happened, Jim reached out with his left hand—the hand he cannot consciously control—and punched Larry—punched him hard—right in the nose.

Participants then rated their agreement to a series of statements on a scale of 1–6, where 1 indicated “strongly agree” and 6 indicated “strongly disagree.”

### 3.2. Participants

Participants were recruited through Amazon's Mechanical Turk. 144 participants (90 male, 54 female) saw one of four vignettes.

### 3.3. Results

A 2 ×  2 analysis of variance (ANOVA) test was performed on participant responses to *Free Will*. I found significant main effects for Conscious Self, F(1, 144) = 536.07, p <  .001, partial eta squared = .793, as well as Deep Self, F(1, 144) = 20.55, p <  .001, partial eta squared = .128. There was no significant interaction (p = .20). The mean responses by vignette were as follows: *Conscious*/*Concordant* M = 5.80, SD = .41; *Conscious*/*Discordant* M = 5.37, SD = .77; *Nonconscious*/*Concordant* M = 2.91, SD = .83; and *Nonconscious*/*Discordant* M = 2.14, SD = 1.06.

The Deep Self condition significantly impacted responses to *Moral Responsibility*, *Blame*, and *Decided*. Participants tended to judge that violent Jim—whose actions were concordant with his Deep Self—was morally responsible (M = 4.03, SD = 1.09) and worthy of blame (M = 4.23, SD = 1.02), though they withheld such judgments from non-violent Jim (M = 3.37, SD = 1.24 and M = 3.20, SD = 1.16, respectively). Further, they were closer to the midpoint of the scale in judging that Jim had decided to punch Larry (M = 3.06, SD = .98 versus M = 2.03, SD = .99). In all three cases, the difference is statistically significant (*Moral Responsibility*: t(67) = 2.34, p = .022; *Blame*: t(67) = 4.04, p <  .001; and *Decided*: t(67) = 4.35, p <  .001).^5^

### 3.4. Discussion

Notably, unlike in experiment 1, in this experiment the Deep Self condition had a significant impact on participant judgments. How should we understand the influence of the Deep Self here? I find the data reported concerning participants who read nonconscious vignettes telling. When the agent's nonconscious action was concordant with his Deep Self, participants tended to say that Jim was morally responsible and that he deserved to be blamed (though they withheld a similar judgment regarding his free will, M = 2.91). Furthermore, they gave a significantly higher rating of agreement with the statement that Jim decided to punch Larry—a statement that seems, in my view, obviously false. It is thus plausible that the description of Jim's violent character contributed to a *motivation* to blame Jim, and this motivation significantly biased responses in the observed direction (see Alicke, 2000).

Although the Deep Self condition had a significant impact, its effect size was much smaller than that of the Conscious Self condition (partial eta squared = .793 versus .128, respectively). It thus appears that, as experiment 1 tentatively indicated, the impact of consciousness on participant attributions of free will is both independent of considerations often taken to motivate Deep Self Views, as well as comparatively much stronger.

Proponents of Deep Self Views may not like this interpretation and suggest that things are not so straightforward regarding this experiment. In particular, such a proponent might argue that the nonconscious vignettes provide no evidence against a Deep Self View because in these vignettes the agent exercises no control at all. If this is true, then—supposing, as many do, that free action requires control^6^—the agent does not act freely.

It is false that Jim exercises no control in punching Larry—the action is not random, it is situationally appropriate and requires a fair amount of motor control to execute. But perhaps the thought is that though Jim's anarchic hand punches Larry, *Jim* does not. The vignette emphasized that Jim's anarchic hand is sometimes at odds with Jim's (presumably conscious) purposes. Participants might judge that the hand is no part of Jim, and thus that Jim does not act freely because Jim does not act. Perhaps some participants would endorse this line of thought, but it is not clear all would. It is equally possible—and consistent with the vignette—to maintain that Jim had some control over his anarchic hand, and that the chief problem here was that Jim lacked *conscious* control. Even so, it seems that more empirical work is needed to be clear about what is driving participant judgments here.

Having made this concession, I hasten to add that I am not interpreting this study as a counterexample to Deep Self Views. The right interpretation, in my view, is that consciousness is central to folk views of free and responsible action, and that the way in which it is central is not captured by extant Deep Self Views. That Deep Self Views neglect the role of consciousness is a blind spot.

## 4. Experiment 3

### 4.1. Procedure

The first two experiments emphasized action production via conscious processes. But it is possible that the importance of consciousness for free will and moral responsibility is not an entirely causal matter. This experiment sought to test whether the absence of consciousness impacts folk attributions of free will or moral responsibility even when the relevant behavior is causally identical to that produced by conscious states and processes.

Similar to experiments 1 and 2, this experiment utilized a 2 (Conscious: nonconscious versus conscious) ×  2 (Action Valence: good versus bad) design. Participants saw one of four vignettes, after which they rated their agreement to a series of statements on a scale of 1–6, where 1 indicated “strongly agree” and 6 indicated “strongly disagree.”

All participants first read the following paragraph:

In the future, humans develop the technology to construct humanoid machines. These machines have very sophisticated computers instead of brains, and very intricate movement-generation systems instead of bones, ligaments and muscles. In fact, they are so sophisticated that they look, talk, and act just like humans, and they integrate into human society with no problem at all. The only way to tell if they are a humanoid machine instead of a human being is to look inside of them (by x-ray, for example).

Then participants read a paragraph that sorted them into a conscious or a nonconscious condition:

**Conscious**

These creations are behaviorally just like human beings, and in addition, these creations possess consciousness. They *actually feel* pain, *experience* emotions, *see* colors, and *consciously* deliberate about what to do.

**Nonconscious**

These creations are behaviorally just like human beings. But, these creations do not possess consciousness. They do not *actually feel* pain (even when they say ‘Ouch!’), they do not *experience* emotions, they do not *see* colors, and they do not *consciously* deliberate about what to do.

Next, participants read a paragraph that sorted them into either a bad action or a good action condition:

**Good**

One day a conscious [nonconscious] humanoid machine named Sal sat at a coffee shop. A man at a nearby table got up to leave, and Sal noticed that the man had left his wallet on the table. As the man walked out the door, Sal quickly walked over to the table, took the wallet, and returned it to the man.

**Bad**

One day a conscious [nonconscious] humanoid machine named Sal sat at a coffee shop. A man at a nearby table got up to leave, and Sal noticed that the man had left his wallet on the table. As the man walked out the door, Sal quickly walked over to the table and stole the wallet.

Participants then rated their agreement with the following statements, on a 1–6 scale:

**Free Will**

When Sal stole [returned] the wallet, he acted of his own free will.

**Moral Responsibility**

Sal is morally responsible for stealing [returning] the wallet.

**Control**

When Sal stole [returned] the wallet, he was in control of his behavior.

**Blame**

Sal deserves to be blamed [praised] for stealing the wallet.

**Decided**

Sal decided to steal [return] the wallet.

**Conceivable**

It makes sense to suppose that though Sal is not conscious, he is behaviorally just like a human being. [It makes sense to suppose that Sal is conscious, and that he is behaviorally just like a human being.]

### 4.2. Participants

Participants were recruited through Amazon's Mechanical Turk. 179 adults (106 male, 73 female) saw one of four vignettes.

### 4.3. Results

A 2 ×  2 analysis of variance (ANOVA) test was performed on participant responses to *Free Will*. I found a significant main effect for the Conscious condition, F(1, 179) = 90.23, p <  .001, partial eta squared = .34, no effect for the Action Valence condition (p = .71), and no interaction (p = .94). The means indicated that participants attributed free will to the conscious humanoid (M = 4.98, SD = 1.25) but not to the nonconscious humanoid (M = 2.97, SD = 1.53). The mean responses (and patterns of significance) to all other statements save *Conceivable* closely tracked those to *Free Will*, and are not reported here.

Given the massive philosophical literature surrounding the conceivability of zombies—that is, creatures who are functionally identical to human beings but without consciousness—I wanted to know whether participants found the nonconscious humanoid conceivable.^7^ Most of them did. Regarding the nonconscious humanoid, 68/94 (73%) answered 4 or higher to *Conceivable*. The mean response was 4.06 (SD = 1.01). Even so, a sizeable minority found Sal inconceivable—for those invested in the philosophical literature on conceivability, an interesting result. By contrast, 76/83 (or 92% of) participants regarded the conscious humanoid as conceivable. The mean response to *Conceivable* for the conscious humanoid was 4.99 (SD = 1.01). The difference between the two means is statistically significant (t(175) = 4.90, p <  .001, two-tailed). It appears that it is more difficult to conceive of the humanoid as nonconscious than as conscious.

### 4.4. Discussion

The data reported here indicate a very strong connection between consciousness on the one hand and free will and moral responsibility on the other. In this experiment, I compared behaviorally identical agents, varying only the presence of consciousness. The difference in attributions of free will (and related notions, such as moral responsibility and the question of whether an agent decided to do as he or she did) is striking. When consciousness was present, participants easily attributed free will (and related notions) to a conscious humanoid (M = 4.98). When consciousness was absent, they withheld these attributions (M = 2.97).

In section 1, I drew a connection between Reasons-Responsive Views of free will and moral responsibility, and the results of this study. The connection is indirect, but I think worth noting. Reasons-Responsive Views seek to account for free will and moral responsibility in terms of an agent's capacity to recognize and respond to reasons for action. Arguably, whatever capacities human beings have in this connection would be shared by the nonconscious humanoids, since the humanoids are behaviorally identical to actual human beings. So it is arguable that Reasons-Responsive Views are missing something important about free will and responsibility: namely, the role of consciousness.

That said, it is possible that consciousness is important for reasons-responding for non-functional reasons. To my knowledge no Reasons-Responsive theorist argues that this is so, but such arguments could be developed. I mention one such argument in the next section.

## 5. What Has Consciousness to Do With Free Will and Moral Responsibility?

The studies reported here indicate that according to most non-philosophers, consciousness is somehow important for free will and moral responsibility. These results do not indicate how we ought to make sense of the purported connection between consciousness on the one hand and free will and moral responsibility on the other. That remains an undone and interesting project. In my view, it is one to which both experimentalists and traditional philosophers of mind and action are well positioned to contribute. In the next two subsections, I consider the prospects for future experimental and philosophical work in this connection.

### 5.1. Consciousness, Free Will, and Moral Responsibility: Experimental Prospects

The extant data indicate that most people find consciousness crucial for free will and moral responsibility. They do not clearly indicate *why* most people do. Figuring that out is a task for future experimental work. How should such work proceed?

I have no special wisdom here, but a few observations seem apt. Consciousness is a complicated phenomenon—a wide range of experience-types and capacities populate our conscious mental lives. Future empirical work might profitably explore connections between particular aspects of consciousness and various aspects of agency. For example, previous experimental work has uncovered close connections between mental activities such as deliberating and deciding and free will. In a study that asked participants to offer their own autobiographical accounts of free action, Stillman, Baumeister, and Mele found that “accounts of free acts were significantly more likely than the accounts of unfree acts to indicate that the person engaged in conscious reflection prior to the action” (2011, p. 391). One possibility is that the folk take the conscious experience of deliberating and deciding to be constitutive of events of deliberation and decision—or perhaps, as experiment 3 suggests, the folk simply find it difficult to conceive of nonconscious deliberation and decision—and that this explains (at least a part of) the connection between consciousness and free will and moral responsibility.

Another important empirical issue concerns the folk's understanding of consciousness. The experiments reported in sections 2–4 emphasize elements of *phenomenal* consciousness, that is, a kind of conscious experience *E* such that “there is something it is like” for a subject to have *E*. But the above experiments also emphasize elements such as deliberation, and the relationship of deliberation to phenomenal consciousness is unclear. Arguably, phenomenal consciousness is not the only concept of consciousness that is relevant to the consciousness-free will connection. Some seem to think access consciousness, or something like it, is more relevant than phenomenal consciousness (Levy, 2014; Schlosser, 2013). This is an explicitly functionalized conception of consciousness. According to Block (1995), a mental state is access conscious if it is poised to be used as a premise in reasoning, and poised for use in control over action and speech. Whether access consciousness is a legitimate concept of *consciousness*, as opposed to something else, is a matter of some dispute. What is important for present purposes is whether the folk distinguish between these concepts of consciousness, whether they run them together, and how this relates to folk views on free will and moral responsibility.

Recent work in experimental philosophy has begun to explore commonsense understandings of consciousness. Sytsma and Machery, for example, have presented results indicating that laypeople do not type mental states as philosophers do—“people do not seem to conceptualize their subjective life as phenomenal” (2010, p. 324). Buckwalter and Phelan (2013) have challenged this interpretation of the Sytsma and Machery results, arguing instead that philosophers and non-philosophers alike may have a robust conception of phenomenal consciousness. There is not space to further explore the experimental philosophy of consciousness here. The present point is simply to note that work in this area is relevant to the question at hand—that is, the connection between consciousness and free will and moral responsibility. Future experimental work on this connection would do well to pay attention to how people conceive of their conscious mental lives.

### 5.2. Consciousness, Free Will, and Moral Responsibility: Philosophical Prospects

A philosopher interested in the consciousness-free will connection might engage in two kinds of project. The first project is broadly negative, and involves arguing against a consciousness-free will connection. This negative project might involve very little reference to the folk—it might consist simply of the development of negative arguments. But the philosopher engaged in this project might also wish to build an error theory regarding (at least some part of) the folk understanding of the consciousness-free will connection.

The second project is broadly positive, and involves building an account of the consciousness-free will connection. This might involve developing arguments in support of folk views, such as they are. But as with the negative project, this positive project might depart from psychological findings regarding how the folk understand the consciousness-free will connection. It is possible to build a positive account of the connection, while arguing that the folk conception of it is mistaken in certain ways. A philosopher might thus engage in elements of the positive and the negative project simultaneously.

Given the focus of this paper, it is worth considering how a Deep Self or a Reasons-Responsive theorist might approach these issues. Consider, first, a Reasons-Responsive theorist. It would be nice for him or her if consciousness is somehow functionally critical for the capacity to recognize and respond to reasons. Perhaps, for example, consciousness is critical for the kind of flexible consideration of reasons that characterizes deliberation in adult human beings (Hodgson, 2012). If so, then the Reasons-Responsive theorist will be able to make the case that his or her view captures what is intuitive about a consciousness-free will connection. Of course, it is unclear whether consciousness is critical in this way—some have argued that consciousness makes little contribution to our executive capacities in general (Rosenthal, 2008). Further, if my interpretation of experiment 3 is right, we might worry that no merely functional account of consciousness's importance could fully capture folk views of the consciousness-free will connection. So the Reasons-Responsive theorist has work to do in order to make such a case.

Alternately, a Reasons-Responsive theorist may argue that consciousness is conceptually critical for the capacity to recognize and respond to reasons. For reasons independent of free will and moral responsibility, Smithies (2012) has recently argued that (phenomenal) consciousness is necessary for the kind of cognitive mental life adult humans enjoy—the kind that involves, e.g., weighing reasons for action in deliberation. Smithies defends what he calls the *rational connection thesis*: “An intentional state plays a rational role if and only if it is either conscious or individuated in such a way that its content is accessible to consciousness as the content of a conscious state” (2012, p. 358). There is not space here to cover Smithies argument for this thesis. I mention it here to note that even if consciousness is not functionally critical for reasons-responding, the Reasons-Responsive theorist has options. Taking sides with Smithies is one.

Deep Self theorists will probably be interested in the Reasons-Responsive project—they agree that recognizing and responding to reasons is important for free will. But Deep Self theorists might also be interested in developing arguments in favor of claims like this one: Apart from its importance (or not) for reasons-responsiveness, consciousness is important for the features of an agent's psychology that constitute the Deep Self. Something like this view has been recently developed by Levy (2014). Levy argues that consciousness is necessary for “the expression of evaluative agency” (2014, p. 88).^8^ For Levy, nonconscious attitudes do not have the right functional profile to appropriately express evaluative agency. Very roughly, according to Levy, nonconscious attitudes are not functionally and inferentially integrated in the right kind of way to the kinds of attitudes (i.e., conscious attitudes) that constitute reasons for us. Nonconscious attitudes cannot express the agent's Deep Self, and as such an agent is exempted from responsibility for the actions such attitudes cause.

This is clearly a Deep Self-friendly understanding of the relation of consciousness to free will and moral responsibility. If Levy is on the right track, then the Deep Self theorist may be able to capitalize on Levy's account to argue that a Deep Self View best captures the folk understanding of the consciousness-free will connection. Alternately, the Deep Self theorist may wish to develop an account of the consciousness-free will connection that does not rest, as Levy's account does, on an empirically-based account of the difference between conscious and nonconscious attitudes. As with the Reasons-Responsive theorist, it is open to the Deep Self theorist to develop arguments that ground a consciousness-free will connection in non-functional considerations.^9^

## 6. Conclusion

I have offered evidence that many laypeople regard consciousness as important to free will and moral responsibility. In sections 2 and 3, I contrasted action production via conscious states and processes with action in concordance with an agent's long-standing and endorsed motivations, values, and character traits. Results indicate that conscious action production is considered much more important for free will than is concordance with motivations, values, and character traits. In section 4, I contrasted the absence or presence of consciousness in behaviorally identical agents. Most participants attributed free will to conscious agents, but not to nonconscious agents.

Given their antecedent commitment to taking folk views seriously, this presents philosophers of free will and moral responsibility with the following explanatory burden: Either develop a substantive theory of the connection between consciousness on the one hand and free will and moral responsibility on the other, or offer justification for jettisoning this seemingly central part of our commonsense understanding of free will and moral responsibility.

## Footnotes

^[1]^ One way to do this is to appeal to elements of common sense that one's view preserves. But this does not seem to me the only way to justify departures from common sense.

^[2]^ Free will and moral responsibility might come apart. But in aiming to capture “the kind of free will required for moral responsibility,” proponents of these theories tie the notions together in a certain way. I omit this qualification in the text, speaking interchangeably of free will and moral responsibility.

^[3]^ Among others, Levy includes Arpaly (2002), Scanlon (1998), Sher (2009), and Smith (2005).

^[4]^ An anonymous referee notes that it is odd that I did not ask this question of participants in the conscious condition. I agree: I assumed that answers to this question in the conscious condition would be at ceiling since participants are explicitly told the answer to it in that condition. But—especially in light of the responses to this statement in the nonconscious condition—it would be nice to have data on whether participants understand awareness of an action as different from “conscious awareness” as emphasized in the vignette. Future research should be more explicit about this issue.

^[5]^ An anonymous referee argues that the lower p-values for the Blame and Decided statements as compared to that for the Moral Responsibility statement call for explanation. If the p-value for Blame was at the same level, I would think that this difference indicated a residual desire to blame Jim even in the nonconscious condition. But this explanation is not available. So I do not know why the p-value for Moral Responsibility is not as low as the others. It is possible that this is a statistical aberration.

^[6]^ Most philosophers find this claim uncontroversial regarding free will. A similar claim regarding moral responsibility is more controversial. Scanlon (1998) and Smith (2005), for example, argue that one can be responsible for attitudes over which one exercises no control.

^[7]^ The humanoids are not strictly speaking philosophical zombies, since those zombies are also physically identical to human beings.

^[8]^ The kind of consciousness at issue for Levy is not phenomenal consciousness. Rather, it involves a kind of ready availability of information for use in reasoning. See Levy (2014, chapter 2).

^[9]^ This does not exhaust the theoretical landscape—a purported consciousness-free will connection is interesting apart from disputes between these two kinds of views, and there is room for theorists not invested in either theory to develop accounts of the connection. I leave it to future work to discern which kinds of account are most promising.
